# Disrupting LIN28 in atypical teratoid rhabdoid tumors reveals the importance of the mitogen activated protein kinase pathway as a therapeutic target

**DOI:** 10.18632/oncotarget.3078

**Published:** 2014-12-26

**Authors:** Melanie F. Weingart, Jacquelyn J. Roth, Marianne Hutt-Cabezas, Tracy M. Busse, Harpreet Kaur, Antoinette Price, Rachael Maynard, Jeffrey Rubens, Isabella Taylor, Xing-gang Mao, Jingying Xu, Yasumichi Kuwahara, Sariah J. Allen, Anat Erdreich-Epstein, Bernard E. Weissman, Brent A. Orr, Charles G. Eberhart, Jaclyn A. Biegel, Eric H. Raabe

**Affiliations:** ^1^ Division of Neuropathology and Sidney Kimmel Comprehensive Cancer Center, Johns Hopkins University, Baltimore, MD, USA; ^2^ Division of Pediatric Oncology, Johns Hopkins University, Baltimore, MD, USA; ^3^ Lineberger Comprehensive Cancer Center, University of North Carolina, Chapel Hill, NC, USA; ^4^ Division of Hematology, Oncology, and Blood & Bone Marrow Transplant, Children's Hospital Los Angeles, Los Angeles, CA, USA; ^5^ The Keck School of Medicine of the University of Southern California, Los Angeles, CA, USA; ^6^ Department of Pathology, St. Jude Children's Research Hospital, Memphis, TN, USA; ^7^ Department of Pediatrics, The Children's Hospital of Philadelphia, Philadelphia, PA, USA; ^8^ Department of Pathology and Laboratory Medicine, The Children's Hospital of Philadelphia, Philadelphia, PA, USA; ^9^ Perelman School of Medicine at the University of Pennsylvania, Philadelphia, PA, USA

**Keywords:** ERK, AZD6244, let-7, RAS, LIN28, malignant rhabdoid tumor

## Abstract

Atypical teratoid rhabdoid tumor (AT/RT) is among the most fatal of all pediatric brain tumors. Aside from loss of function mutations in the *SMARCB1* (*BAF47/INI1/SNF5)* chromatin remodeling gene, little is known of other molecular drivers of AT/RT. LIN28A and LIN28B are stem cell factors that regulate thousands of RNAs and are expressed in aggressive cancers. We identified high-levels of LIN28A and LIN28B in AT/RT primary tumors and cell lines, with corresponding low levels of the LIN28-regulated microRNAs of the *let-7* family. Knockdown of LIN28A by lentiviral shRNA in the AT/RT cell lines CHLA-06-ATRT and BT37 inhibited growth, cell proliferation and colony formation and induced apoptosis. Suppression of LIN28A in orthotopic xenograft models led to a more than doubling of median survival compared to empty vector controls (48 vs 115 days). LIN28A knockdown led to increased expression of *let-7b* and *let-7g* microRNAs and a down-regulation of *KRAS* mRNA. AT/RT primary tumors expressed increased mitogen activated protein (MAP) kinase pathway activity, and the MEK inhibitor selumetinib (AZD6244) decreased AT/RT growth and increased apoptosis. These data implicate LIN28/RAS/MAP kinase as key drivers of AT/RT tumorigenesis and indicate that targeting this pathway may be a therapeutic option in this aggressive pediatric malignancy.

## INTRODUCTION

Atypical teratoid rhabdoid tumor (AT/RT) is one of the most aggressive pediatric tumors of the central nervous system [1]. Despite the use of intensive multimodality treatment, the overall survival rate is less than 50 percent [2]. Due to its aggressive nature and resistance to existing treatment, there is a significant need for novel therapeutic targets in AT/RT.

Deletions and mutations of the *SMARCB1* (*BAF47/INI1/SNF5)* gene are the hallmark of AT/RT tumors, yet there are no other recurrent genetic abnormalities [3-5]. SMARCB1 is a component of the SWI/SNF chromatin remodeling complex, and loss of function of SMARCB1 can dysregulate thousands of genes across the genome [6]. While mutations at this locus are the defining genetic alteration of AT/RT, much of the biology contributing to the development and aggressiveness of AT/RT is poorly understood [7].

Another distinguishing characteristic of AT/RT is a heterogeneous histologic appearance with characteristics of several distinct cell lineages, suggesting that stem cells may play a role in the tumorigenesis of AT/RT [1]. Primary AT/RT tumors express stem cell factors such as SAL4, KLF4, and MUSHASHI [8]. LIN28A is a stem cell factor that was first discovered as a regulator of development, and it has since been implicated in multiple aggressive cancers [9]. *LIN28A* expression is high in embryonic stem cells and maintains pluripotency by inhibiting *let-7* induced differentiation [10]. The *let-7* family of microRNAs in turn acts as tumor suppressors, through inhibition of oncogenes including *KRAS*, *HMGA2*, and *MYC*. We have previously identified LIN28A as a driver of proliferation and invasion in adult and pediatric glioblastoma [11]. As part of a screen of a series of pediatric brain tumors for LIN28A expression, we identified high level LIN28A expression in AT/RT samples by immunohistochemistry. We hypothesized that LIN28A and LIN28B might contribute to AT/RT tumorigenicity by upregulating at the RNA level many oncogenic proteins which contribute to AT/RT growth, invasion, and metabolism. We report that LIN28A and LIN28B are highly expressed in AT/RT primary tumors and cell lines, and that knockdown of LIN28A suppresses AT/RT growth and tumorigenicity and leads to downregulation of *KRAS* mRNA. Examination of AT/RT primary tumor tissue shows expression of the RAS effector phospho-ERK in 77% of AT/RT. We demonstrate that the MEK inhibitor selumetinib also suppresses AT/RT cell line growth and induces apoptosis, phenocopying LIN28A loss. These results indicate activation of a LIN28/RAS/MAP kinase pathway in AT/RT and suggest that MEK inhibitors may be effective in this tumor.

## RESULTS

### LIN28A and LIN28B are highly expressed in primary AT/RT tumor samples and cell lines

**Fig. 1:**
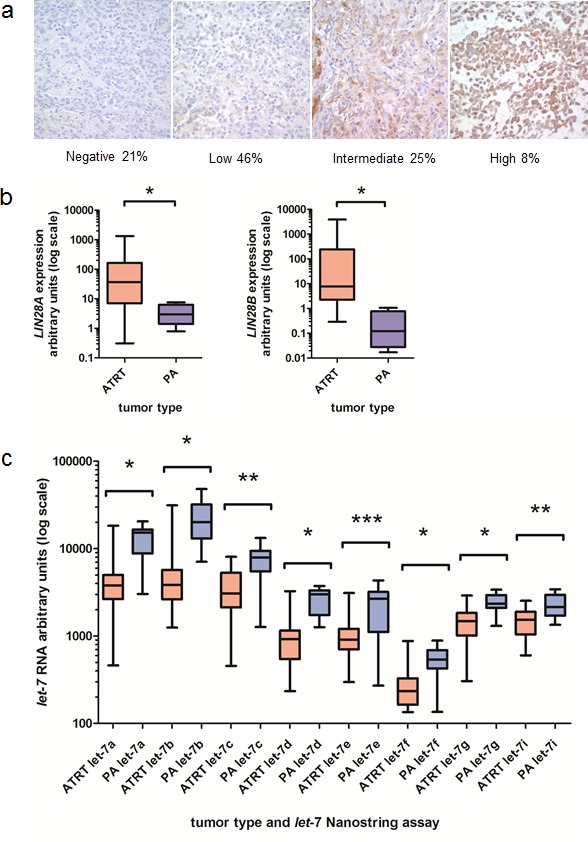
Increased expression of *LIN28A* and *LIN28B* and decreased *let-7* expression in AT/RT A. LIN28A immunohistochemistry performed on 24 AT/RT primary tumors revealed high-level LIN28A expression in more than 30 percent of AT/RT (moderate plus high staining). Normal brain did not show any LIN28A staining. B. We identified increased *LIN28A* and *LIN28B* mRNA in AT/RT compared to pediatric pilocytic astrocytoma (PA) samples by qPCR. For *LIN28A*, we compared 29 AT/RT primary tumors to 11 PA samples. For *LIN28B*, we compared 23 AT/RT tumors to 7 PA samples. Data is presented in a box and whisker plot showing the median and range of expression of *LIN28A* (left) and *LIN28B* (right). Note the log scale. Asterisk indicates p=0.0002 for *LIN28A* and p=0.0006 for *LIN28B* by Mann-Whitney test. C. AT/RT express decreased amounts of *let-7* species compared to pediatric PA samples as measured by Nanostring assay. Note the log scale. * indicates p<0.0001, ** indicates p<0.001, *** indicates p<0.01 by Mann-Whitney test. For each *let-7* Nanostring assay, we compared 26 AT/RT tumors to 18 PA tumors.

We have previously identified high-level expression of LIN28A and LIN28B in approximately 30 percent of pediatric and adult glioblastoma [11]. LIN28B is expressed in a poor-prognosis subset of medulloblastoma, [12] and a subtype of primitive neuroectodermal tumors has increased expression of LIN28A [13, 14]. By immunohistochemistry (IHC), we found that 19/24 AT/RT were positive for LIN28A (79%). Approximately 30% of AT/RT have moderate or high-level LIN28A staining (Figure 1A). Normal cortex did not show any LIN28A expression by IHC (Supplemental Figure 1A). We were not able to interrogate AT/RT at the level of histology for LIN28B, due to a lack of LIN28B antibodies validated for IHC. We next investigated the mRNA expression of *LIN28A* and *LIN28B* in AT/RT tumors by qPCR, using pediatric pilocytic astrocytoma (PA) as a comparator. These tumor types have not been systematically interrogated for LIN28 expression and have relatively few genomic alterations. We found that primary AT/RT tumors expressed high level *LIN28A* and *LIN28B* mRNA (Figure 1B, p=0.002 for *LIN28A* and p=0.006 for *LIN28B* by Mann-Whitney test) compared to PA tumors. Of the 23 AT/RT tumors that were evaluated by qPCR for both *LIN28A* and *LIN28B* expression, 6 tumors had predominantly high *LIN28A* expression, 5 tumors had predominantly high *LIN28B* expression, 7 tumors had equivalent high-level expression of both *LIN28A* and *LIN28B*, and 5 tumors had low-level expression of *LIN28A* and *LIN28B,* comparable to levels found in PA. In total, 18/23 AT/RT (78%) showed increased expression of *LIN28A*, *LIN28B* or both.

LIN28A is known to negatively regulate the tumor-suppressing microRNAs of the *let-7* family, and when we performed Nanostring assay for *let-7* family members, we found that AT/RT tumors had significantly lower *let-7* levels than PA samples (Figure 1C, p<0.01 for all *let-7* species by Mann-Whitney test).

When we examined medulloblastoma primary tumors for comparison of LIN28A expression, we found that 24 percent of these aggressive pediatric brain malignancies (15/63 tumors) expressed increased levels of LIN28A by IHC compared to normal brain (Supplemental Figure 1A). By qPCR we found increased *LIN28B* expression in 16/31 (52%) of medulloblastoma samples, compared to normal pediatric cerebellum (Supplemental Figure 1B). This is consistent with previous reports of increased *LIN28B* expression in medulloblastoma [12].

### AT/RT cell lines express high levels of LIN28A and LIN28B

**Fig. 2:**
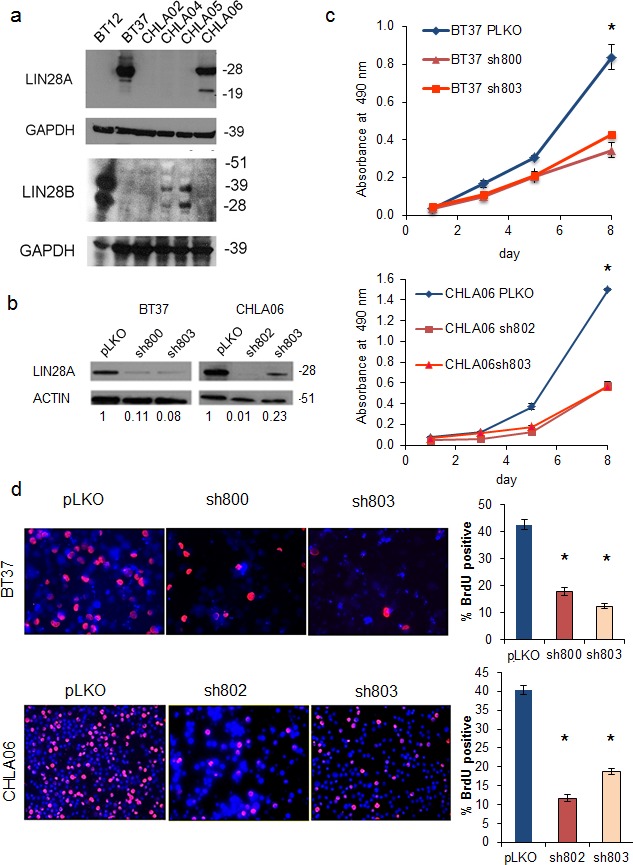
Lentiviral short hairpin RNA knockdown of LIN28A suppresses AT/RT growth and proliferation A. Western blot showing increased expression of LIN28A (top) and LIN28B (bottom) in AT/RT cell lines. CHLA02 expresses *LIN28A* by qPCR, but at a level that is not detectable by western blot (data not shown). B. Western blot showing knockdown of LIN28A in AT/RT cell lines BT37 and CHLA-06 after lentiviral infection with two different shRNA constructs. pLKO is a control, empty-vector construct. Numbers under the blot indicate the relative change in intensity of LIN28A band, normalized to ACTIN, as measured by densitometry. C. MTS growth assay showing suppression of growth by knockdown of LIN28A in BT37 (top) and CHLA-06 (bottom). Asterisk = p<0.005 by *t*-test comparing each shRNA to pLKO. D. Suppression of proliferation as measured by BrdU incorporation after knockdown of LIN28A in BT37 and CHLA-06. At left is representative immunofluorescence showing decreased BrdU incorporation. At right is a graph showing quantification of percent BrdU positivity. Asterisk = p<0.0005 by *t*-test comparing each shRNA to pLKO.

We found that of the 6 AT/RT cell lines in our laboratory, 2 expressed LIN28A at high level by western blot, and 3 expressed LIN28B at high level (Figure 2A). By qPCR, we detected *LIN28A* in CHLA-02, indicating that in this panel, 100% of AT/RT express *LIN28A* or *LIN28B* (data not shown).

### Knockdown of LIN28A inhibits AT/RT growth and proliferation

To examine the importance of LIN28A in the tumorigenicity of AT/RT, we used short hairpin RNA constructs packaged in lentivirus to inhibit LIN28A protein expression. Effective LIN28A knockdown by two distinct shRNA constructs was confirmed by western blot (Figure 2B) in two cell lines derived from primary human AT/RT tumors (BT37 and CHLA-06).

Knockdown of LIN28A resulted in a significant reduction of growth in both BT37 and CHLA-06. Compared to pLKO empty vector, in BT37 we detected a 50% reduction in growth at day 8 (Figure 2C, p=0.003 for sh800 vs pLKO control and p=0.004 for sh803 vs pLKO, by Student's *t*-test). Similarly, in CHLA-06 we detected a 60% inhibition in growth with LIN28A knockdown by MTS assay (Figure 2C, p=0.0004 for sh802 vs pLKO control and p=0.009 for sh803 vs pLKO, by Student's *t*-test). In both BT37 and CHLA-06 LIN28A-shRNA cells, we observed a 65% reduction in the percentage of proliferating cells after LIN28A knockdown, as measured by BrdU incorporation, compared to pLKO empty vector controls (Figure 2D, for BT37, p=0.0004 for sh800 vs pLKO, p=0.00008 for sh803 vs pLKO; CHLA-06, p=0.00005 for sh802 vs pLKO, p=0.0001 for sh803 vs pLKO by Student's *t-*test).

### Knockdown of LIN28A suppresses AT/RT clonogenic growth and tumorigenicity

**Fig. 3:**
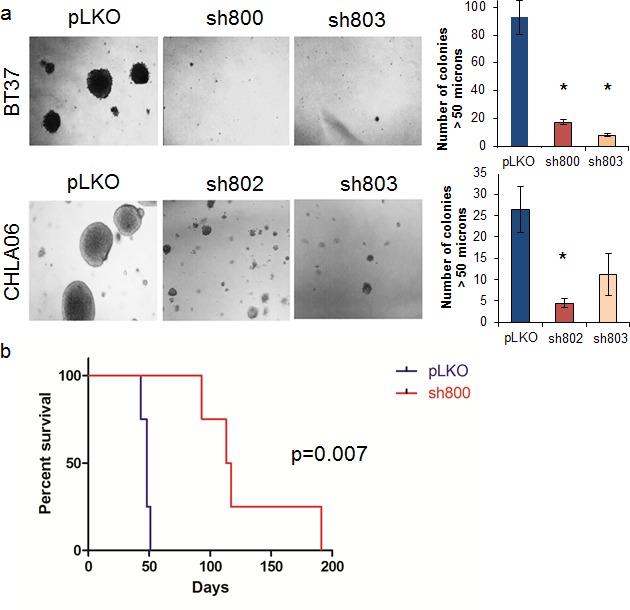
Knockdown of LIN28A suppresses AT/RT clonogenicity and tumorigenicity A. Photomicrograph (100x) showing colony formation after LIN28A knockdown in CHLA-06 and BT37. Right is quantification of colonies greater than 50 μM. Asterisk = p<0.01 by Student's *t*-test comparing shRNA to pLKO control. For CHLA-06 sh803 vs pLKO, p = 0.06. B. Kaplan-Meier curve showing survival after injection of BT37 cells into the deep gray matter of immunodeficient mice. The median survival of mice (n=4 in each group) injected with BT37 cells with LIN28A knockdown was 115 days compared to 48 days for mice injected with BT37 pLKO control transduced cells (p=0.007 by log rank test).

To assess the role of LIN28A in the clonogenicity of AT/RT cell lines, LIN28-shRNA transduced cells and pLKO control cells were plated as single cells in soft agar and allowed to grow for 2 weeks. Compared to control, colonies formed by BT37 and CHLA-06 cells with LIN28A knockdown were reduced by between 50 and 90 percent (Figure 3A – For BT37 p=0.0002 sh800 vs pLKO, p=0.0001 sh803 vs pLKO; for CHLA-06 p= 0.009 sh802 vs pLKO, p=0.06 sh803 vs pLKO). We further tested the ability of LIN28A deficient AT/RT cells to form tumors by injecting shLIN28A-transduced or pLKO control transduced BT37 cells into brains of immunodeficient mice. Within two months of injection, all mice injected with BT37 pLKO control had died from malignant tumors (Figure 3B). The median survival of mice (n=4 in each group) injected with BT37 cells with LIN28A knockdown was 115 days compared to 48 days for mice injected with BT37 pLKO control transduced cells (p=0.007 by log rank test). There was no significant difference in microscopic appearance of the tumors that formed in either group, and we determined by LIN28A immunohistochemistry that LIN28A knockdown tumors expressed LIN28A at a level equal to that found in pLKO tumors (data not shown).

### Suppression of LIN28 induces apoptosis in AT/RT cell lines

**Fig. 4:**
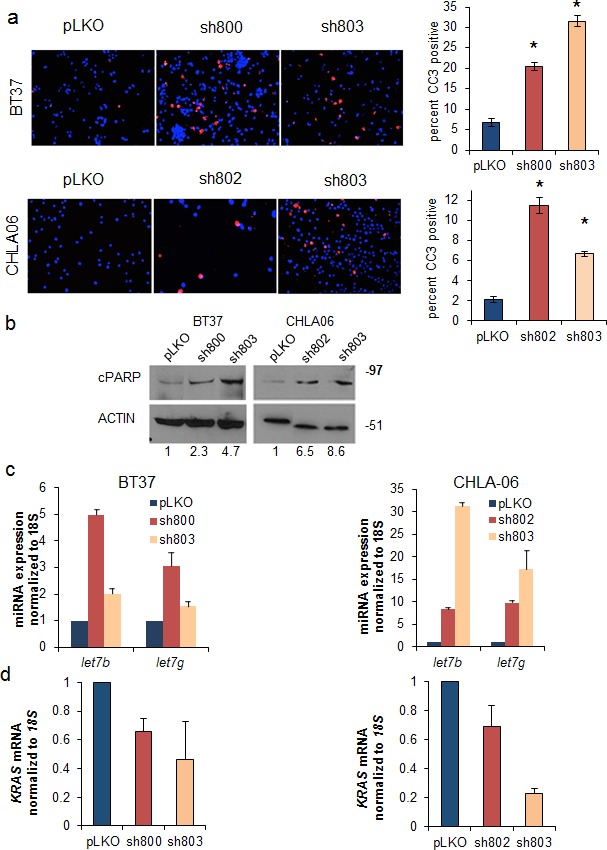
Suppression of LIN28A leads to upregulation of *let-7* microRNAs, induction of apoptosis, and downregulation of *KRAS* A. (Left) Knockdown of LIN28A leads to apoptosis as measured by cleaved caspase 3 (CC3) immunofluorescence in BT37 (top) and CHLA-06 (bottom) cells. Bar graphs (right) show quantification of 3 separate experiments. Asterisk = p<0.01 by Student's t-test comparing each shRNA to pLKO B. Western blot showing upregulation of cleaved PARP (cPARP), an indicator of apoptosis, after LIN28A knockdown in BT37 (left) and CHLA-06 (right). Numbers under the blot indicate the relative change in cPARP intensity, normalized to ACTIN, as measured by densitometry. C. LIN28A knockdown leads to upregulation of *let-7b* and *let-7g* as measured by qPCR in BT37 (left) and CHLA-06 (right). Results are from a representative experiment, which was repeated with similar results. Error bars represent the standard deviations of the representative experiment. D. Reduced expression of *KRAS* as measured by qPCR after LIN28A knockdown in BT37 (left) and CHLA-06 (right). Graphs represent averaged results from two separate experiments. Error bars represent the standard deviations of all qPCR wells for each condition (N=6).

We investigated the mechanism by which loss of LIN28A suppressed the growth of AT/RT cell lines. We found that LIN28A knockdown in BT37 and CHLA-06 cells led to a 4 to 6 fold increase in the percentage of cells expressing cleaved caspase 3 (CC3) compared to controls, as measured by immunofluorescence (Figure 4A – For BT37 p=0.0005 sh800 vs pLKO and p=0.0001 for sh803 vs pLKO; for CHLA-06 p=0.004 sh802 vs pLKO and p=0.009 sh803 vs pLKO). We also observed an induction in the expression of cleaved PARP (cPARP) protein in CHLA-06 and BT37 cells with LIN28A knockdown by western blot (Figure 4B). Levels of cell death were further assessed with cell cycle analysis, and we observed an increase in the sub-G1 population in both BT37 and CHLA-06 LIN28A-shRNA cells compared to controls (Supplemental Figure 2).

### Knockdown of LIN28A results in an upregulation of *let-7* micro-RNAs and suppression of *KRAS*

We next interrogated the effect of LIN28A knockdown on the tumor suppressing *let-7* microRNAs. We observed an increase in the expression of *let-7g* and *let-7b* after LIN28A knockdown in both BT37 and CHLA-06 cells compared to pLKO empty vector controls (Figure 4C). We assessed the effect of LIN28A knockdown on potential targets of the *let-7* miRNAs by qPCR. Although we did not detect a decrease in the expression of *MYC*, *HMGA2* or *IGF2* (data not shown), we did observe a reduction in the expression of *KRAS* mRNA in BT37 and CHLA-06 cells with LIN28A knockdown (Figure 4D). Suppression of LIN28A expression led to decreased levels of phospho-ERK in CHLA-06 cells (Supplemental Figure 3).

### AT/RT primary tumors show high level phospho-ERK activation

**Fig. 5:**
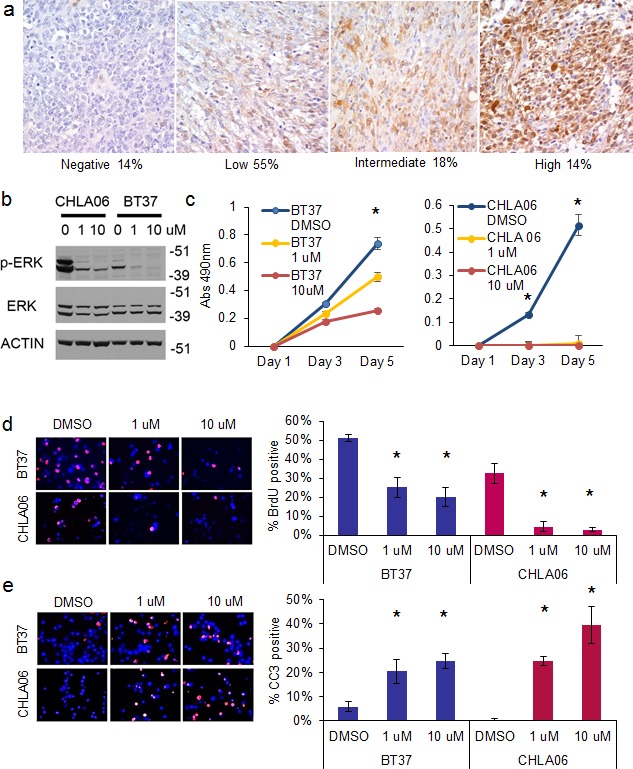
Inhibition of the MAP kinase pathway with MEK inhibitor selumetinib suppresses the growth and proliferation of AT/RT cells and induces apoptosis A. Immunohistochemistry showing high expression of phospho-ERK in AT/RT. Representative 400X photomicrographs of phospho-ERK staining on an AT/RT primary tumor tissue microarray containing 22 evaluable tumors. Tumor scored as no staining on the left, followed by low, intermediate, and high staining. Below the images is indicated the percentage of the total number of AT/RT tumors that fell into the respective intensity. B. Western blot showing that selumetinib efficiently inhibits ERK phosphorylation in AT/RT. C. MTS plot showing that selumetinib inhibits the growth of BT37 (left) and CHLA-06 (right). Asterisk = p<0.01 by *t-*test, compared to DMSO treated cells. D. Immunofluorescence showing decreased BrdU incorporation in BT37 and CHLA-06 cells after treatment with selumetinib. Quantification of BrdU incorporation is at right. Asterisk = p<0.01 by *t*-test comparing selumetinib to DMSO control. E. Induction of apoptosis after selumetinib treatment as measured by cleaved caspase 3 (CC3) immunofluorescence. Quantification of BrdU incorporation is at right. Asterisk = p<0.05 by *t*-test comparing selumetinib to DMSO control.

We hypothesized that the RAS pathway may be important for AT/RT growth. We determined by immunohistochemistry on 22 primary human AT/RT tumors that 86% of AT/RT expressed phospho-ERK, a key readout of MAP kinase pathway activation (Figure 5A). While some tumors had diffuse high-level staining for phospho-ERK, others had more patchy immunoreactivity. Of those showing activated ERK, 37% (7 out of 19 tumors) showed intermediate or high level expression. There was a positive correlation between pERK and LIN28A expression (R=0.57, p=0.009 by Spearman correlation).

### Targeting the MAP kinase pathway suppresses AT/RT growth and induces apoptosis

We disrupted the RAS/MAP kinase pathway using selumetinib, a MEK inhibitor in phase I and II clinical trials in children with brain tumors (NCT01089101). Selumetinib efficiently blocked ERK activation in AT/RT cell lines (Figure 5B) using standard doses [15]. Treatment with selumetinib for 5 days suppressed the growth of BT37 and CHLA-06 (Figure 5C, for BT37 p=0.003 for 1 uM and p=0.001 for 10 uM vs DMSO control; for CHLA-06 p=0.009 for 1 uM and 10uM vs DMSO control by *t-*test). We obtained similar results using the LIN28B-expressing BT12 AT/RT cell line (p=0.00009 for 10 uM vs DMSO control by *t*-test) (Supplemental Figure 4).

We observed a 50 percent or greater decrease in proliferation, as measured by BrdU incorporation, after 4 days of treatment with selumetinib in both BT37 and CHLA-06 cells (Figure 5D – for BT37 p=0.008 for 1uM and p=0.004 for 10 uM vs DMSO; for CHLA-06 p=0.005 for 1uM and p=0.009 for 10 uM vs DMSO by *t-*test). MEK inhibition caused a quadrupling or more of apoptosis in both AT/RT cell lines, as measured by CC3 immunofluorescence after 4 days of selumetinib treatment (Figure 5E - for BT37 p=0.02 for 1uM and p=0.001 for 10 uM vs DMSO; for CHLA-06 p=0.001 for 1uM and p=0.01 for 10 uM vs DMSO by *t-*test). Levels of cell death were further assessed with cell cycle analysis, and we observed an increase in the sub-G1 population in both BT37 and CHLA-06 after treatment with selumetinib (Supplemental Figure 5).

## DISCUSSION

A greater understanding of molecular abnormalities contributing to the development of AT/RT is essential to the improvement of therapeutic options. However, multiple genome-wide analyses have yielded no reports of consistent genetic aberrations in addition to mutations in the *SMARCB1* gene [3, 4]. Our study investigated the molecular biology underlying the tumorigenicity of AT/RT.

We found frequent overexpression of the cell-reprogramming factors *LIN28A* and *LIN28B* in AT/RT primary tumor samples and cell lines. In total, 18/23 (78%) of AT/RT tumors showed increased expression of *LIN28A* and/or *LIN28B* by qPCR. Our data together with recent reports of increased HMGA2 expression in AT/RT suggest a broad reliance on the LIN28/HMGA2 pathway in this tumor [16].

Increased expression of LIN28 and HMGA2 are consistent with reports of the role of this pathway in other aggressive tumors [9]. In brain tumors, we recently reported that LIN28A promotes invasion and tumorigenesis in glioblastoma in part through upregulation of HMGA2 [11]. LIN28B expression is a poor prognostic factor in medulloblastoma [12], LIN28A is associated with worse prognosis in primitive neuro-ectodermal tumor [13, 14], and LIN28A and LIN28B are known to be expressed in germ cell tumors [17]. Our finding of high-level LIN28A expression in 30 percent of AT/RT is consistent with results of other groups who have performed IHC on AT/RT for LIN28A [18-20]. The experiments described here are the first to our knowledge to investigate the functional significance of the LIN28 pathway in AT/RT and indicate that LIN28 is important for the overall growth, proliferation, clonogenicity and tumorigenicity of this aggressive tumor.

In orthotopic xenograft experiments, we found that the BT37 tumors that formed after LIN28A knockdown had morphological characteristics and LIN28A expression that was indistinguishable from pLKO-transduced cells. The increased latency of tumor formation that we identified after LIN28A knockdown was likely due to the vast majority of LIN28A knockdown cells being unable to grow. The tumors that did form were likely derived from a small population of BT37 cells that escaped from LIN28A knockdown. The universal expression of LIN28A in the BT37 pLKO and LIN28A knockdown tumors further supports the importance of LIN28 in AT/RT.

On a mechanistic level, LIN28A is known to regulate the *let-7* family of microRNAs [21, 22]. Downregulation of *let-7* has also been linked to human malignancies [23-27]. In our LIN28A knockdown experiments, we found LIN28A levels to be inversely correlated with *let-7b* and *let-7g* microRNA expression. We also found downregulation of *KRAS* mRNA and phospho-ERK signaling after LIN28A suppression. Identification of the RAS pathway as highly active in AT/RT, and that MEK inhibition suppresses the growth of AT/RT cells and induces apoptosis is consistent with recent findings of dysregulation of cyclin dependent kinases (CDKs) in AT/RT and an increasingly recognized role of LIN28 in regulating CDKs [28]. The frequent lack of expression of p16^INK4a^ in AT/RT [29] is also consistent with an ability to tolerate high-level MAP kinase signaling and avoid oncogene induced senescence [30].

The broad expression of p-ERK suggests that MEK may be a therapeutic target in AT/RT. We found equal responses to the MEK-inhibitor selumetinib in BT37 and CHLA06 (LIN28A-expressing) and BT12 (LIN28B-expressing) cell lines, indicating that both LIN28A and LIN28B-expressing AT/RT may be sensitive to this class of pharmaceutical. Selumetinib is currently in phase I/II clinical trials in pediatric patients with low grade gliomas (NCT01089101). Additional, more potent, MEK inhibitors with improved brain penetration are also being tested in early phase trials in children (NCT02124772).

In AT/RT there is a significant absence of mutations in canonical pathways normally implicated in aggressive cancer [4]. However, both SMARCB1 and LIN28A/B regulate large portions of the genome through chromatin remodeling and RNA stabilization respectively [31, 32]. A recent study reported that LIN28 regulates more than 3,000 different RNA species, many of which are implicated in oncogenic pathways [33]. LIN28A and SMARCB1 can suppress senescence by regulating cell proliferation and growth pathways [6]. LIN28 is also able to adjust the metabolism of cells, promoting tumor growth and survival [34-36]. AT/RT is known to express multiple stem cell markers, and may arise from stem cells [8, 20]. Persistent expression of LIN28 and other stem cell factors may be due to the cell of origin of AT/RT. SMARCB1 loss may lead to a block of differentiation and persistence of a stem-like state. The ability of LIN28 and SMARCB1 to simultaneously regulate large sections of the genome and suppress senescence could spark the development of a malignant tumor such as AT/RT.

In summary, our study demonstrates the importance of the LIN28/*let-7* pathway in AT/RT. Knockdown of LIN28A using short hairpin RNA constructs resulted in a significant decrease in cell growth, proliferation, and clonogenic potential, as well as a corresponding induction of apoptosis. In orthotopic xenograft experiments, loss of LIN28A was sufficient to increase the latency of AT/RT tumor formation in immunodeficient mice. Investigation of LIN28A/*let-7* targets in AT/RT demonstrated downregulation of *KRAS* after LIN28A knockdown, and we demonstrated the upregulation of *KRAS* downstream effectors by identifying phospho-ERK signaling in primary AT/RT. Inhibition of MEK by selumetinib in AT/RT led to growth suppression and induction of apoptosis, indicating that this pathway may be a promising target for therapeutic intervention in this currently highly lethal tumor.

## METHODS

### Cell Culture

The AT/RT cell lines CHLA-02-ATRT, CHLA-04-ATRT, and CHLA-06-ATRT have been previously described [37]. CHLA-02-ATRT and CHLA-04-ATRT are available from ATCC (Manassas, VA). The BT37 AT/RT cell line was derived from a serially passaged xenograft derived from a patient with AT/RT. Cells were grown as semi-adherent cultures in RPMI/10% FBS media (500 mL RPMI, 50 mL fetal bovine serum, 1% L-glutamine, 1% Penicillin/Streptomycin, Life Technologies, Grand Island, NY). Cells were split at high density after scraping and gentle titration. BT12 cells (available through the Children's Oncology Group cell repository, Lubbock, TX) were grown in RPMI/10% FBS media (500 mL RPMI, 50 mL fetal bovine serum, 1% L-glutamine, 1% Penicillin/Streptomycin. Selumetinib (AZD6244) was obtained from Selleck Chemicals (Houston, TX) and dissolved in DMSO.

### Viral infections and short hairpin constructs

Lentivirus vectors encoding short hairpin RNA constructs against LIN28A were purchased from Sigma-Aldrich (St. Louis, MO) (TRCN0000021800, TRCN0000021802 and TRCN00000803). To produce the required lentiviral particles, 293T cells were transfected with VSVG envelope plasmid, delta 8.9 gag/pol plasmid and the plasmid of interest using Fugene transfection reagent (Promega, Madison, WI) per the manufacturer's instructions, as described previously [11]. Supernatants were then collected at 48 hours, 72 hours, and 96 hours and stored at 4 C. For infection of AT/RT cell lines, adherent cells and neurospheres were dissociated into single cells with gentle titration and Accutase (Sigma-Aldrich), respectively, and incubated with the lentivirus containing LIN28A-shRNA, or pLKO empty vector. After 48 hours in culture, infected cells were selected using 1 ug/ml puromycin. All experiments were performed within 14 days of infection of cells.

### Immunofluorescence assays

For immunofluorescence assays, cells were cytospun onto positively charged slides (ThermoFisher Scientific, Waltham, MA), washed once with PBS, and fixed with 4% paraformaldehyde for 15 minutes. After washing three times with PBST, cells were permeabilized with 0.1% Triton/PBS, blocked for 15 minutes with 5% normal goat serum/PBST, and then incubated with the cleaved caspase 3 primary antibody (Cell Signaling Technology, Danvers, MA). Cells were then washed three times with PBST and incubated for 45 minutes in the dark with the Cy-3 conjugated secondary antibody (Jackson Immunoresearch, West Grove, PA). Following this incubation, cells were counterstained with DAPI and mounted with anti-fade mounting media (Vectastain, Burlingame, CA).

### Flow cytometry

Cells were fixed in ice cold 70% ethanol and processed according to the Guava cell cycle flow cytometry protocol (EMD Millipore, Billerica, MA). Data were captured on a Guava flow cytometer.

### Growth Assays

Bromodeoxyuridine incorporation assays were performed by incubating cells with 100 uM 5-bromo-2′-deoxyuridine (BrdU, Sigma-Aldrich) for 6 hours. Cells were taken out of the BrdU medium, dissociated into single cells with gentle titration and Accutase, washed with PBS, and fixed with methanol overnight at 4 C. Cells were cytospun onto positively charged slides and processed as described above. Anti-BrdU antibody was used as per the manufacturer's direction (Sigma-Aldrich, B2531) at 1:500 dilution and visualized as described above. Results were analyzed in Adobe Photoshop, where BrdU-positive cells were counted using the count tool. To assess relative growth, cells were plated in 96-well plates in triplicate at densities of 4000 cells per well. Relative cell number was then measured at 0, 2, 4, and 7 days using the colorimetric CellTiter 96 MTS assay (Promega).

### Colony formation assay in soft agarose

Six-well plates were coated with a bottom agar/media mixture, which was made from a 1:1 mixture of a prepared 2x concentration of neurosphere media and 1 percent melted agarose (Life Technologies) in water. Cells were incubated in Accutase (Sigma-Aldrich), triturated into single cells, and placed into a top agarose/media mixture (0.7%) an immediately plated into the 6-well plates at a density of 20,000 cells/well in 1.5 mL of agarose. 1.5 ml of media was then placed into each well. Fresh media was added every 7 days, and the colonies were grown for 1.5 to 2 weeks. Colonies were then visualized by staining with nitroblue tetrazolium (Sigma-Aldrich) overnight at 37 C and quantified using MCID Elite software (Cambridge, England, UK) with a gate diameter of 50 microns.

### Quantitative RT-PCR

Relative RNA expression was analyzed by real-time PCR analysis in triplicate with SYBER Green reagents (Applied Biosystems, Foster City, CA) per manufacturer's instructions on an I-Cycler IQ5 real-time detection system (Bio-Rad, Hercules, CA). Expression levels were determined using the delta/delta CT method and then normalized to either *β-ACTIN* or *18S* rRNA. Primer sequences were as follows: human *LIN28A* forward: CGGGCATCTGTAAGT, reverse: CAGACCCTTGGCTGA; human *β-ACTIN* forward: CCCAGCACAATGAAGATCAA, reverse: GATCCACACGGAGTACTTG; human *18S* forward: GTAACCCGTTGAACCCCATT, reverse: CCATCCAATCGGTAGTAGCG; human *KRAS* forward: GGGGAGGGCTTTCTTTGTGT, reverse: GTCCTGAGCCTGTTTTGTGTC. Relative expression of microRNAs, mature *let-7a, b*, and *g* were quantified using Taqman MicroRNA Assay (Life Technologies) per the manufacturer's instructions. Expression levels were normalized to *18S* rRNA.

### Western blotting

Western blots were performed as previously described [11]. Specific antibodies were used as per the manufacturer's instructions: LIN28A (Cell Signaling Technologies), β-ACTIN (Santa Cruz Biotechnology, Inc., Dallas, TX), GAPDH; Fitzgerald Industries, Acton, MA).

### Intracranial xenograft tumors

For animal care and anesthesia, “Principles of laboratory animal care” (NIH publication No. 86-23, revised 1985) were followed, using a protocol approved by the Johns Hopkins Animal Care and Use Committee, in compliance with the United States Animal Welfare Act regulations and Public Health Service Policy. Intracranial xenografts were produced in anesthetized animals as previously described [11]. Injection guide holes were produced by an 18-gauge beveled needle and 1×10^5^ viable cells were injected in 5 ul of growth medium into the right striatum stereotactically through a needle connected to a Hamilton syringe. Cells were injected using the following coordinates: antero-posterior = −3mm; medio-latral=2 mm; dorso-ventral =3 mm. Animals were sacrificed upon signs of distress suggestive of an intracranial mass lesion such as neurologic deficits, poor grooming, and cachexia. Xenograft tumors were embedded in paraffin and processed for immunohistochemistry by the Johns Hopkins Histopathology Core.

### Statistical Analysis

Statistical analysis was performed using GraphPad Prism (GraphPad Software, San Diego, California) or Excel (Microsoft, Redmond, WA). All tests were two sided unless indicated otherwise, and p values under 0.05 were considered significant unless otherwise indicated. Error bars on graphs are SEM unless otherwise indicated.

### Primary brain tumor samples

Brain tumor specimens were procured by the departments of pathology at Johns Hopkins University School of Medicine Department of Pathology, the Children's Hospital of Philadelphia, and St. Jude Children's Research Hospital with institutional review board approval. All samples were de-identified.

### Immunohistochemistry

Phospho-ERK antibody (Cell Signaling Technology) was used as described previously [38]. Anti-LIN28A (Cell Signaling Technology) was used as described previously [16]. AT/RT tissue cores were scored by a neuropathologist (CGE) using H-scores (H) (0–200) which were obtained by multiplying the intensity of stain (0: no stain, 1: weak stain, 2: strong stain) by percentage (0–100) of neoplastic cells showing the staining intensity. Tumors were scored for phospho-ERK and LIN28A as being negative (score of 0), low (5 to 45), intermediate (50-95) or high (100 and above).

### LIN28 and *let-7* RNA quantification

Total RNA was extracted from frozen brain tumors using the Qiagen miRNeasy kit (Qiagen, Valencia, CA) according to the manufacturer's instructions. The quantity and quality of the RNA was determined using a Nanodrop 1000 (ThermoFisher Scientific).

Quantitative RT-PCR was used to determine the relative expression of *LIN28A* and *LIN28B* in both primary AT/RT and pilocytic astrocytoma (PA) brain tumor samples. Expression levels were determined using the delta/delta Ct method on the ABI 7500 FAST real-time detection system with the Taqman assays, *LIN28A* – Hs00702808_s1 and *LIN28B* – hs01013729_m1, as per the manufacturer's guidelines (Applied Biosystems). All samples were run in triplicate and *GAPDH* was used as the endogenous control for the normalization.

A total of 45 RNA samples, 26 AT/RT and 18 PA, were submitted to Nanostring for microRNA expression analysis using the microRNA nCounter assay (version 1), which measured the expression level of 654 human microRNAs (Nanostring, Seattle, WA).

The raw Nanostring data were analyzed using the methods described in the nCounter Expression Data Analysis Guide (http://www.nanostring.com/media/pdf/MAN_nCounter_Gene_Expression_Data_Analysis_Guidelines.pdf). In the first round of analysis, the technical reproducibility of the assay was assessed, and one lane from each set of technical replicates was chosen to represent the tumor in the second round of analysis. The steps for the two rounds of analysis consisted of (i) calculating the average value of all negative probes across all tumor samples; (ii) calculating the geometric mean for each sample from the overall top 100 expressing microRNAs; (iii) performing a lane normalization using the normalization factor determined by the average geometric mean for the entire assay; (iv) determining the negative threshold for the assay by calculating the mean +2 standard deviation of the negative control probes for each lane and then averaging these values across the assay.

The Pearson correlation was used at the end of the first round of analysis to determine the technical reproducibility of the assay. A correlation value above 0.95 was considered highly correlated. A random number generator was then used to select the lane that was representative of each tumor in the second round of analysis. The microRNA counts determined at the end of the two rounds of analysis were then used for downstream statistical analyses in GraphPad Prism.

## SUPPLEMENTARY MATERIAL AND FIGURES


