# Supplementary material for: Disrupting LIN28 in atypical teratoid rhabdoid tumors reveals the importance of the mitogen activated protein kinase pathway as a therapeutic target
Source: Oncotarget. 2014 Dec 26;6(5):3165–77. doi: 10.18632/oncotarget.3078 (PMC4413645; doi:10.18632/oncotarget.3078)
Supplement: Supplementary file 1 [file oncotarget-06-3165-s001.pdf]

Disrupting LIN28 in atypical teratoid rhabdoid tumors reveals the importance of the mitogen activated protein kinase pathway as a therapeutic target

Supplementary Material

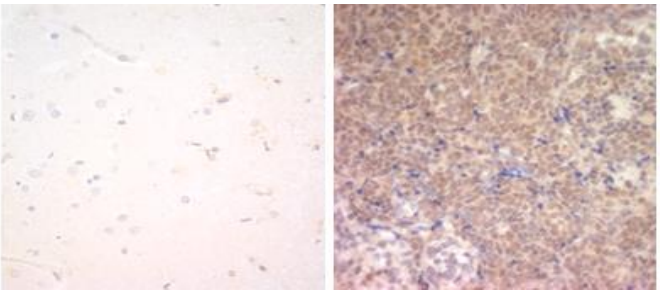

Supplemental Figure 1A: LIN28A is expressed in a subset of medulloblastoma, as detected by immunohistochemistry. Normal brain is largely negative for LIN28A expression (left). In contrast, 24 percent of medulloblastoma (15/63 samples) showed increased expression of LIN28A (right). Magnification 400X.

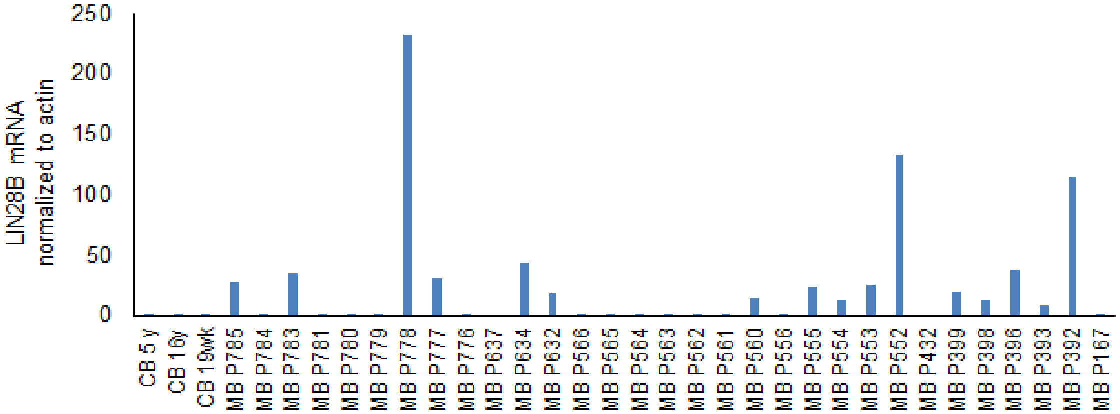

Supplemental Figure 1B: *LIN28B* is expressed in 16/31 (52%) of medulloblastoma samples, compared to developing cerebellum (positive is considered 5-fold increased expression compared to control normal cerebellum 5 year old sample (far left)).

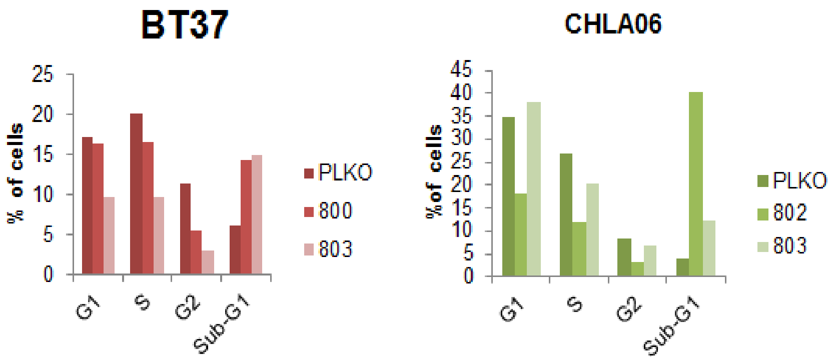

Supplemental Figure 2: Knockdown of LIN28A leads to increased apoptosis (sub G1 fraction) as measured by cell cycle assay using a Guava flow cytometer after propidium iodide staining.

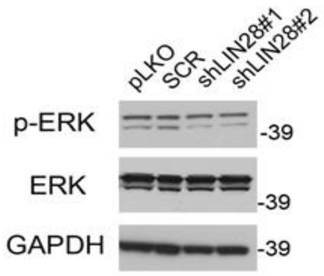

Supplemental Figure 3: Lentiviral shRNA suppression of LIN28A leads to a 50% reduction p42 phospho-ERK expression (lower band in p-ERK blot) in the CHLA-06 AT/RT cell line. Quantification was performed using ImageJ densitometry and normalized to total ERK expression. SCR= scramble control shRNA

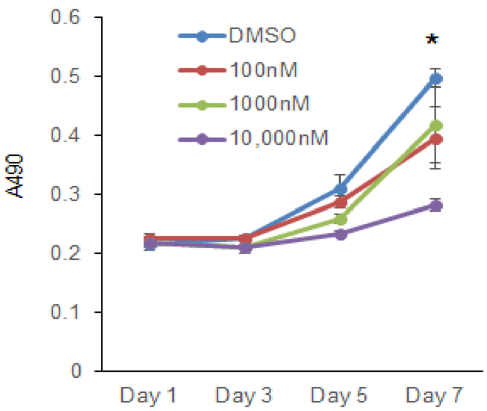

Supplemental Figure 4: Selumetinib treatment leads to decreased growth in the BT 12 AT/RT cell line as measured by MTS assay. Asterisk indicates p<0.0005 10 uM vs DMSO. 100 nM and 1 uM concentrations are not significantly different (p=0.16) at day 7.

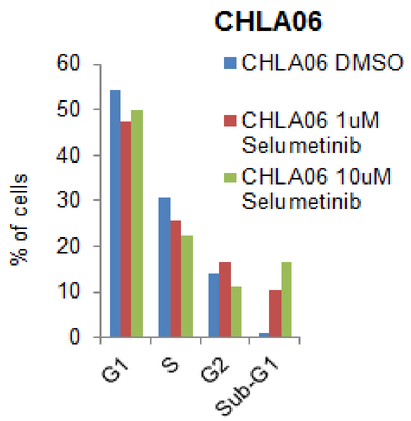

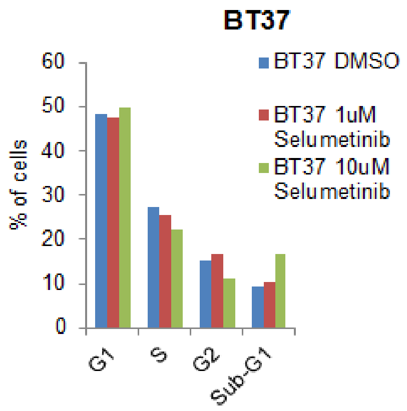

Supplemental Figure 5: Selumetinib treatment leads to decreased S-phase entry and increased apoptosis as measured by cell cycle assay using a Guava flow cytometer after propidium iodide staining.
